# Supplementary material for: Linking Soil Fungal Generality to Tree Richness in Young Subtropical Chinese Forests
Source: Microorganisms. 2019 Nov 10;7(11):547. doi: 10.3390/microorganisms7110547 (PMC6921041; doi:10.3390/microorganisms7110547)
Supplement: Supplementary file 1 [file microorganisms-07-00547-s001.pdf]

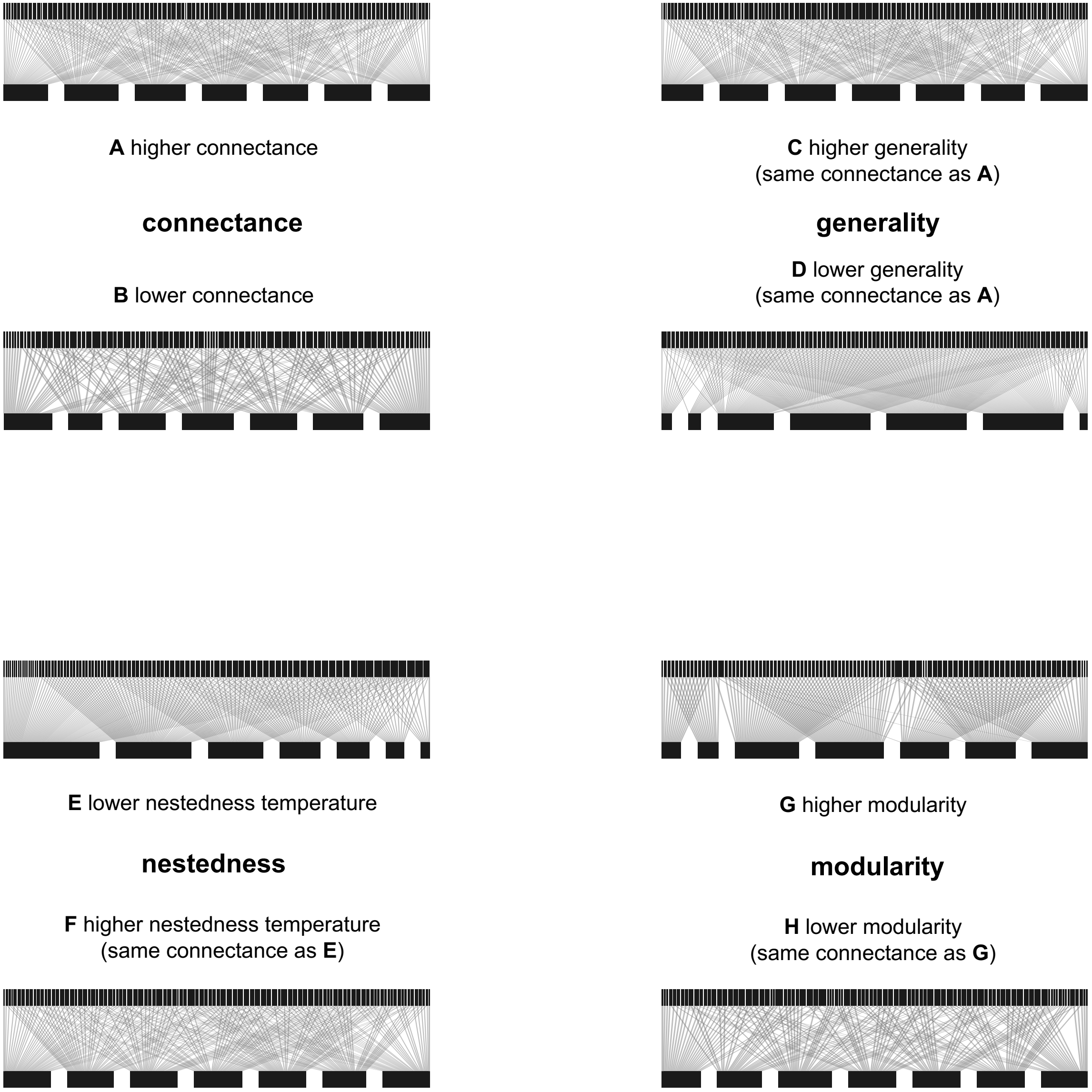


**Figure S1**: Conceptual bipartite networks with equal numbers of higher- and lower level partners, visualizing differences in connectance (A, B), generality (C, D), nestedness (E, F), and modularity (G, H).

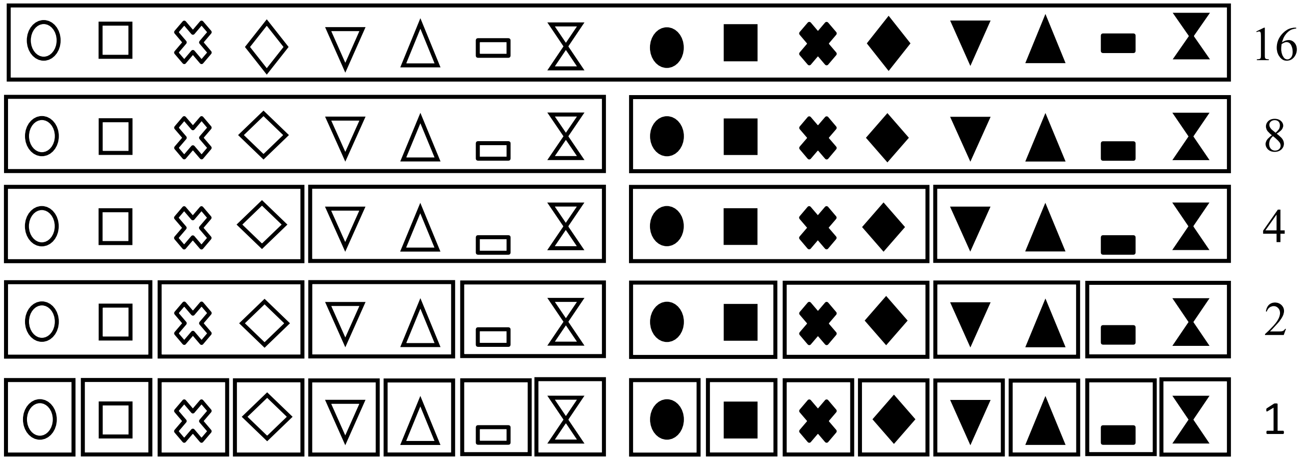


| Tree species | Type | No. | Tree species | Type | No. |
|---|---|---|---|---|---|
| *Castanea henryi* (Skan) Rehd. & Wils. | EcM | 5 x 5 | *Cyclobalanopsis glauca* (Thunb.) Oerst. | EcM | 5 x 5 |
| *Nyssa sinensis* Oliver | AM | 5 x 5 | *Quercus fabri* Hance | EcM | 5 x 5 |
| *Liquidambar formosana* Hance | AM | 5 x 5 | *Rhus chinensis* Mill. | AM | 5 x 5 |
| *Sapindus saponaria* Linn. | AM | 5 x 5 | *Schima superba* Gardner & Champion | AM | 5 x 5 |
| *Choerospondias axillaris* (Roxb.) Burtt & Hill | AM | 5 x 5 | *Castanopsis eyrei* (Champ.) Tutcher/ *C. carlesii* (Hemsl.) Hay. | EcM | 5 x 5 |
| *Triadica sebifera* (L.) Small | AM | 5 x 5 | *Cyclobalanopsis myrsinifolia* (Blume) Oerst. | EcM | 5 x 5 |
| *Quercus serrata* Murray | EcM | 5 x 5 | *Lithocarpus glaber* (Thunb.) Nakai | AM | 5 x 5 |
| *Castanopsis sclerophylla* (Lindl.) Schott. | EcM | 5 x 5 | *Koelreuteria bipinnata* Franch. | AM | 5 x 5 |
| **Number of soil samples** | | **200** | **Number of soil samples** | | **200** |

**Figure S2.** Broken-stick-design of the experimental forest plots. Plot design presented for the **b**iodiversity and **e**cosystem **f**unctioning (BEF) experiment China, study site A. The 16 species mix was sub-divided in two times eight species mixtures. These were likewise partitioned into four, two and one tree species communities. Tree species are shown as symbols. Similarity of symbols was only chosen to emphasize the experimental design and does not imply any similarities or dissimilarities of tree species traits. No.: Number of samples.

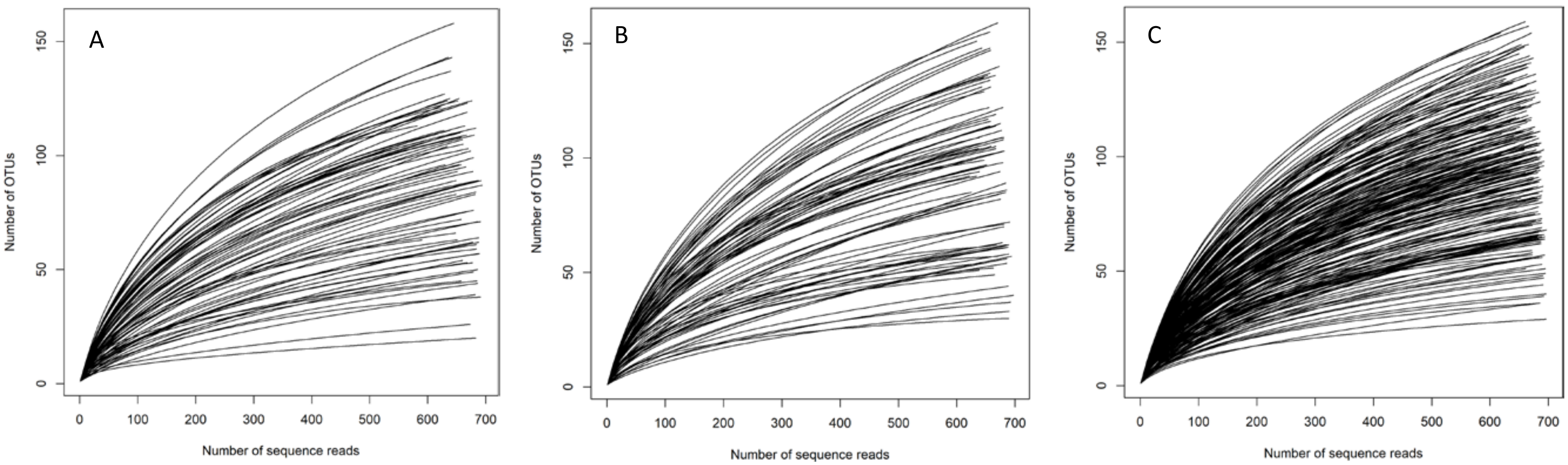


**Figure S3.** Rarefaction curves of monoculture (A) two tree species mixtures (B) high tree diversity mixtures (4, 8 or 16 tree species mixtures).

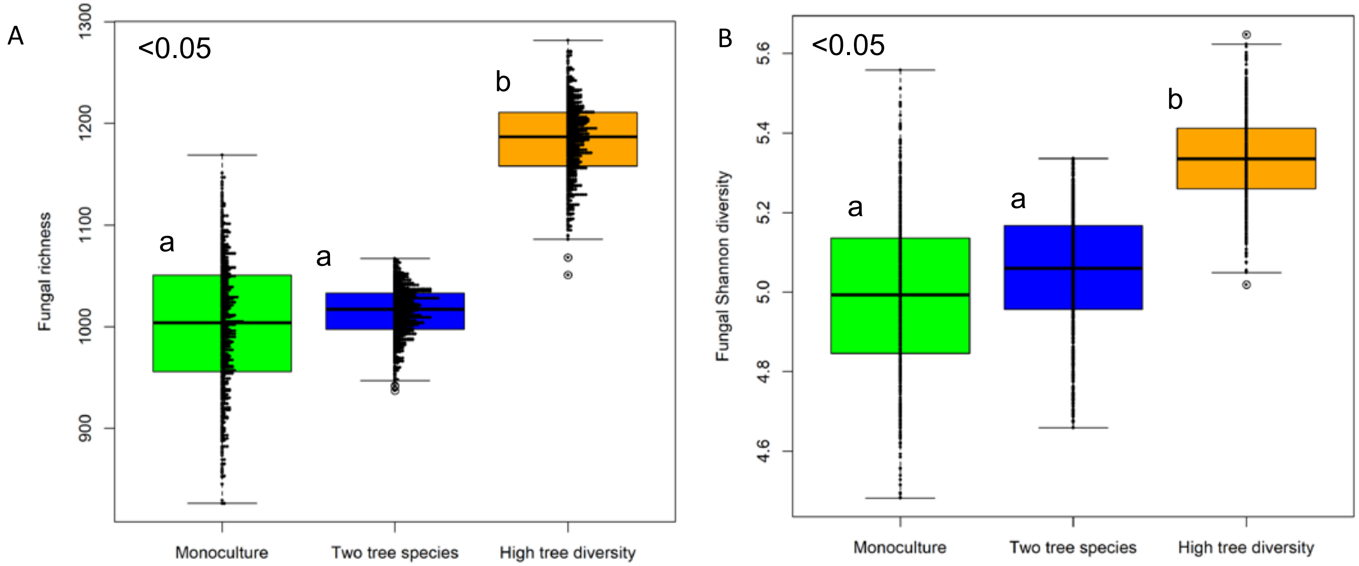


**Figure S4.** Fungal richness (A) and fungal Shannon diversity (B) across the 576 subsampling permutations.

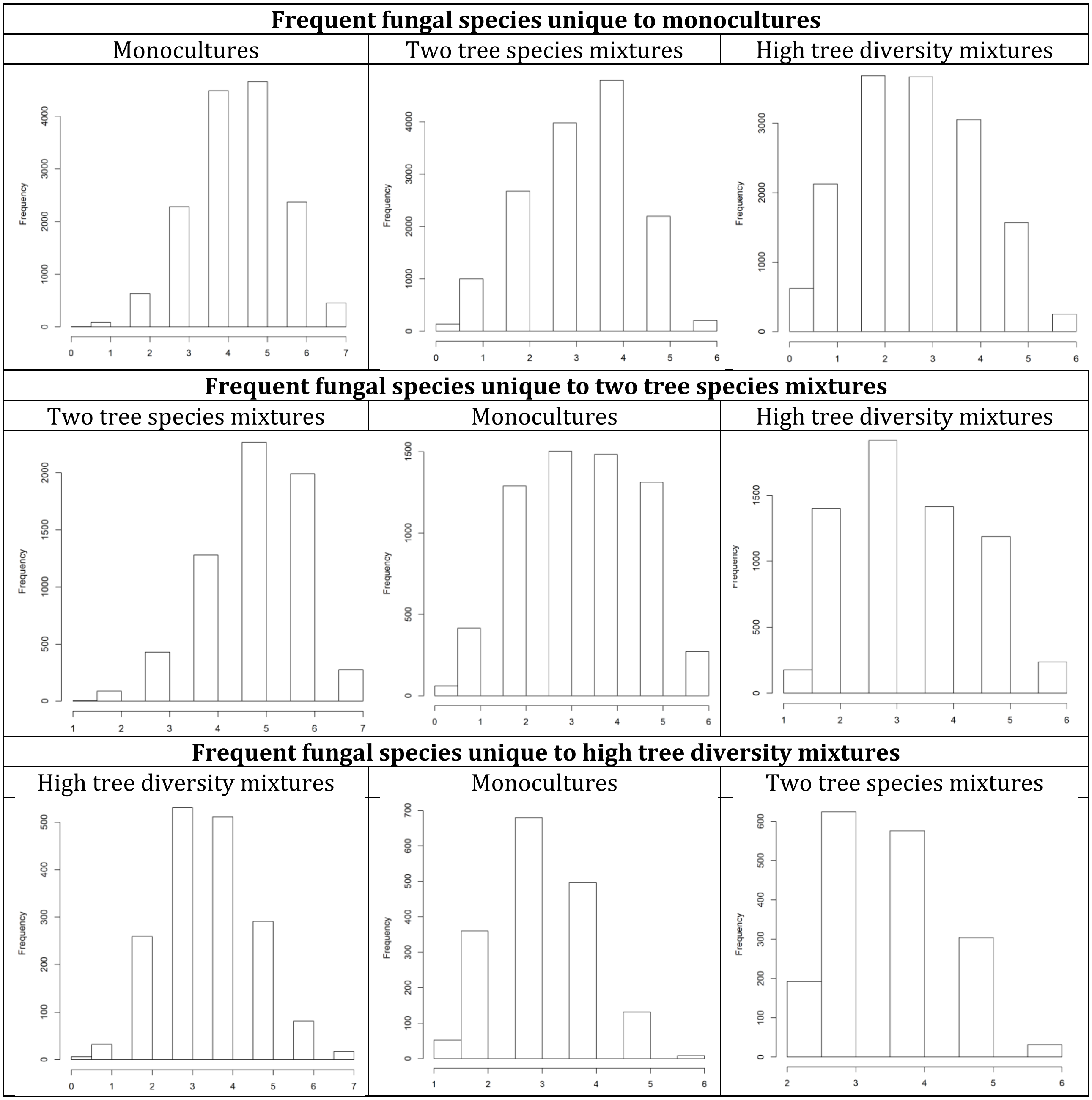


**Figure S5.** Histograms visualizing occurrence patterns of unique frequent fungal OTUs of one tree diversity level across all tree diversity levels. Frequent fungal OTUs were defined as those occurring on all seven plots in at least one permutation.

**Table S1.** Network characteristics for tree-fungal bipartite analysis based on co-occurrence. Network metrics (A) were compared between the tree diversity levels, while network nestedness (B) was compared against a null model. Networks were calculated for all possible link thresholds (one to five out of five samples) in which co-occurrence was observed. The Kruskal-Wallis test was applied to test for a significant difference of each network characteristic, the p value is reported. Median values of the 576 subsampling routines are given for the three tree diversity levels and below stated whether the Kruskal-Wallis test for multiple comparisons was significant (T=TRUE) or not (F=FALSE) for the group comparisons (indicated by numbers).

| A | number OTUs | modularity | connectance | fungal generality | fungal C score | mean shared fungal partners |
|---|---|---|---|---|---|---|
| **1 co-occurrence** | <0.001 | <0.001 | <0.001 | <0.001 | <0.001 | <0.001 |
| <u>Median</u> | | | | | | |
| mono | 1004 | 0.43 | 0.26 | 2.61 | 0.64 | 69.17 |
| two mix. | 1017 | 0.41 | 0.27 | 2.73 | 0.63 | 77.98 |
| high | 1187 | 0.46 | 0.24 | 2.39 | 0.67 | 66.67 |
| 1-2/1-3/2-3 | F/T/T/ | T/T/T/ | T/T/T/ | T/T/T/ | T/T/T/ | T/T/T/ |
| **2 co-occurrences** | <0.001 | <0.001 | <0.001 | <0.001 | <0.001 | <0.001 |
| <u>Median</u> | | | | | | |
| mono | 423 | 0.50 | 0.22 | 2.27 | 0.68 | 20.19 |
| two mix. | 427 | 49 | 0.23 | 2.33 | 0.68 | 21.14 |
| high | 550 | 0.55 | 0.21 | 2.06 | 0.72 | 20.71 |
| 1-2/1-/.2-3 | F/T/T/ | T/T/T/ | T/T/T/ | T/T/T/ | F/T/T/ | T/_F/T/ |
| **3 co-occurrences** | <0.001 | <0.001 | <0.001 | <0.001 | <0.001 | <0.001 |
| <u>Median</u> | | | | | | |
| Mono | 206 | 0.52 | 0.22 | 2.26 | 0.67 | 9.43 |
| two mix. | 198 | 0.51 | 0.22 | 2.33 | 0.68 | 9.57 |
| high | 251 | 0.58 | 0.2 | 2 | 0.74 | 8.48 |
| 1-2/1-3/2-3 | T/T/T/ | F/T/T/ | F/T/T/ | T/T/T/ | F/T/T/ | F/T/T/ |
| **4 co-occurrences** | <0.001 | <0.001 | <0.001 | <0.001 | <0.001 | <0.001 |
| <u>Median</u> | | | | | | |
| mono | 96 | 0.51 | 0.22 | 2.35 | 0.65 | 4.83 |
| two mix. | 83 | 0.49 | 0.23 | 2.53 | 0.63 | 4.86 |
| high | 117 | 0.56 | 0.2 | 2.08 | 0.72 | 4.43 |
| 1-2/1-3/2-3 | T/T/T/ | T/T/T/ | T/T/T/ | T/T/T/ | T/T/T/ | F/T/T/ |
| **5 co-occurrences** | <0.001 | <0.001 | <0.001 | <0.001 | <0.001 | <0.001 |
| <u>Median</u> | | | | | | |
| mono | 45 | 0.52 | 0.22 | 2.33 | 0.66 | 2.19 |
| two mix. | 39 | 0.51 | 0.23 | 2.37 | 0.65 | 2.05 |
| high | 56 | 0.58 | 0.2 | 1.95 | 0.72 | 1.9 |
| 1-2/1-3/2-3 | T/T/T/ | F/T/T/ | T/T/T/ | F/T/T/ | F/T/T/ | T/T/_F/ |

| B | | NODF median | NODF Wilcox.p |
|---|---|---|---|
| 1 **co-occurrence** | Mono | 27.83 | <0.001 |
| | Nullmodel | 56.71 | |
| | Two mix. | 29.42 | <0.001 |
| | Nullmodel | 56.83 | |
| | High | 24.61 | <0.001 |
| | Nullmodel | 56.66 | |
| 2 **co-occurrences** | Mono | 22.18 | <0.001 |
| | Nullmodel | 54.74 | |
| | Two mix. | 23.67 | <0.001 |
| | Nullmodel | 53.87 | |
| | High | 17.8 | <0.001 |
| | Nullmodel | 54.24 | |
| 3 **co-occurrences** | Mono | 21.51 | <0.001 |
| | Nullmodel | 57.6 | |
| | Two mix. | 22.59 | <0.001 |
| | Nullmodel | 57.48 | |
| | High | 15.66 | <0.001 |
| | Nullmodel | 57.32 | |
| **4 co-occurrences** | Mono | 22.91 | <0.001 |
| | Nullmodel | 59.76 | |
| | Two mix. | 26.3 | <0.001 |
| | Nullmodel | 60.04 | |
| | High | 16.85 | <0.001 |
| | Nullmodel | 58.71 | |
| **5 co-occurrences** | Mono | 21.47 | <0.001 |
| | Nullmodel | 59.63 | |
| | Two mix. | 24.25 | <0.001 |
| | Nullmodel | 58.67 | |
| | High | 14.98 | <0.001 |
| | Nullmodel | 59.37 | |

**Network modularity:** A high value indicates stronger connections within than between modules, **Network connectance:** Realized proportion of possible links, **Fungal generality:** Mean effective number of tree species per fungal species, **Mean number of shared fungal partners:** Mean number of fungal species that two tree species interact with, **Fungal C score:** Average degree of co-occurrence for all possible pairs of fungal OTUs. Values close to 1 indicate that there is evidence for disaggregation, e.g. through competition. Value close to 0 indicate aggregation of species (i.e. no repelling forces between species); **NODF:** nestedness metric (**n**estedness metric based on **o**verlap and **d**ecreasing **f**ill.

**Table S2.** Top 20 specialist fungal OTUs identified by maximum phi coefficient values.

| | Fungal_guild | Phylum | Class | Order | Family | Genus |
|---|---|---|---|---|---|---|
| **Otu00046** | Ectomycorrhizal | Basidiomycota | Agaricomycetes | Agaricales | Hymenogastraceae | Hymenogaster |
| **Otu00561** | Ectomycorrhizal | Basidiomycota | Agaricomycetes | Agaricales | Entolomataceae | Entoloma |
| **Otu01026** | Ectomycorrhizal | Basidiomycota | Agaricomycetes | Thelephorales | Thelephoraceae | Tomentella |
| **Otu01059** | Unknown | Rozellomycota | GS11 | NA | NA | NA |
| **Otu01081** | Unknown | NA | NA | NA | NA | NA |
| **Otu01112** | Ectomycorrhizal | Basidiomycota | Agaricomycetes | Thelephorales | Thelephoraceae | Tomentella |
| **Otu01197** | Unknown | Rozellomycota | GS11 | NA | NA | NA |
| **Otu01234** | Orchid Mycorrhizal | Basidiomycota | Agaricomycetes | Cantharellales | Tulasnellaceae | Epulorhiza |
| **Otu01639** | Saprotroph | Ascomycota | Archaeorhizomycetes | Archaeorhizomycetales | Archaeorhizomycetaceae | Archaeorhizomyces |
| **Otu01746** | Unknown | Ascomycota | Leotiomycetes | Helotiales | NA | NA |
| **Otu01872** | Saprotroph | Mucoromycota | NA | Mortierellales | Mortierellaceae | Mortierella |
| **Otu01993** | Ectomycorrhizal | Basidiomycota | Agaricomycetes | Cantharellales | Ceratobasidiaceae | Ceratobasidium |
| **Otu02009** | Unknown | Ascomycota | Eurotiomycetes | NA | NA | NA |
| **Otu02292** | Ectomycorrhizal | Basidiomycota | Agaricomycetes | Thelephorales | Thelephoraceae | Tomentella |
| **Otu02384** | Unknown | Ascomycota | Xylonomycetes | GS34 | NA | NA |
| **Otu02663** | Saprotroph | Basidiomycota | Agaricomycetes | Agaricales | Clavariaceae | Clavaria |
| **Otu02893** | Saprotroph | Ascomycota | Eurotiomycetes | Eurotiales | Aspergillaceae | Penicillium |
| **Otu03239** | Unknown | Ascomycota | NA | NA | NA | NA |
| **Otu03384** | Ectomycorrhizal | Basidiomycota | Agaricomycetes | Agaricales | Entolomataceae | Entoloma |
| **Otu04209** | Ectomycorrhizal | Basidiomycota | Agaricomycetes | Sebacinales | Sebacinaceae | Sebacina |

**Table S3.** Frequent fungal OTUs at all three tree diversity treatments (monocultures, two tree species mixtures and high tree diversity mixtures). Frequent fungal species were defined as fungal OTUs appearing at least once on all seven plots of one subsampling permutation.

| | Fungal_guild | Kingdom | Phylum | Class | Order | Family |
|---|---|---|---|---|---|---|
| Otu00002 | Saprotroph | Fungi | Ascomycota | Archaeorhizomycetes | GS31 | NA |
| Otu00003 | Saprotroph | Fungi | Basidiomycota | Geminibasidiomycetes | Geminibasidiales | Geminibasidiaceae |
| Otu00005 | Saprotroph | Fungi | Ascomycota | Eurotiomycetes | Eurotiales | Trichocomaceae |
| Otu00009 | Saprotroph | Fungi | Ascomycota | Sordariomycetes | Hypocreales | Hypocreaceae |
| Otu00012 | Unknown | Fungi | Ascomycota | Leotiomycetes | Helotiales | NA |
| Otu00019 | Saprotroph | Fungi | Mucoromycota | NA | Mortierellales | Mortierellaceae |
| Otu00034 | Saprotroph | Fungi | Basidiomycota | Agaricomycetes | Trechisporales | Hydnodontaceae |
| Otu00038 | Arbuscular Mycorrhizal | Fungi | Glomeromycota | Glomeromycetes | Glomerales | Glomeraceae |
| Otu00068 | Unknown | Fungi | NA | NA | NA | NA |
| Otu00080 | Plant Pathogen | Fungi | Ascomycota | Dothideomycetes | Capnodiales | Cladosporiaceae |
| Otu00084 | Saprotroph | Fungi | Ascomycota | Sordariomycetes | Hypocreales | Hypocreaceae |
| Otu00203 | Saprotroph | Fungi | Basidiomycota | Agaricomycetes | Agaricales | Clavariaceae |
| Otu00211 | Unknown | Fungi | Basidiomycota | Agaricomycetes | Agaricales | NA |
| Otu00273 | Saprotroph | Fungi | Mucoromycota | NA | Mortierellales | Mortierellaceae |
| Otu00298 | Plant Pathogen | Fungi | Ascomycota | Sordariomycetes | Xylariales | Sporocadaceae |
